# Imprime PGG, a yeast β-glucan immunomodulator, has the potential to repolarize human monocyte-derived M2 macrophages to M1 phenotype

**DOI:** 10.1186/2051-1426-2-S3-P191

**Published:** 2014-11-06

**Authors:** Anissa SH Chan, Xiaohong Qiu, Adria Bykowski Jonas, Myra L Patchen, Nandita Bose

**Affiliations:** 1 Biothera, Eagan, MN, USA

## Background and objective

Imprime PGG (IPGG) has shown promising clinical efficacy in combination with monoclonal antibody treatment of cancer [1]. In mice, complement receptor 3 (CR3) on innate immune cells (neutrophils and macrophages) is required for IPGG's anti-tumor activity [2,3]. In human *in vitro *studies, opsonized IPGG binds CR3 on neutrophils and monocytes, but only on cells from individuals with high levels of endogenous anti-β-glucan antibodies (ABA), which is also a potential biomarker for enhanced clinical response to IPGG [1]. Although IPGG has been shown to prime circulating neutrophils and monocytes, no data is available on its effect on N1/N2 neutrophil or M1/M2 macrophage tissue counterparts that can skew the immunostimulatory *vs*. immunosuppressive balance of the tumor microenvironment. The objective of this study was to investigate effects of IPGG treatment on monocyte differentiation to M1 or M2 macrophages.

## Design and results

**Figure 1:**
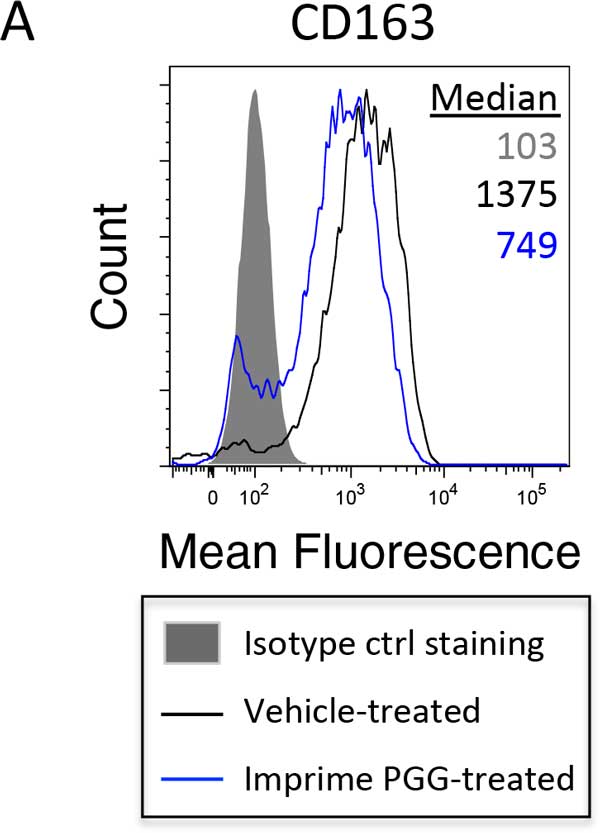
**M2 macophages derived from Imprimer PGG-treated monocytes have reduced expression of CD163, a key M2 marker**. Human whole blood was treated with vehicle or Imprime PGG (25 μf/mL) for 2 hours at 37°C. CD14+ monocytes were then enriched using Ficoll gradient and magnetic bead separation. The cells (5 × 105 cells per mL) were then cultured in either M1-skewing (XVivo 10 media supplemented with 5% autologous serum and 50 ng/mL recombinant human GM-CSF) or M2-skewing (XVivo 10 media supplemented with 10% autologous serum and 100 ng/mL recombinant human M-CSF) conditions for 6 days. Macrophages were harvested and expression of a panel of M1/M2-specific markers was measured by flow cytometry. Shown here is a representative histogram of down-modulation of mean fluorescence intensity of CD163 marker in M" macrophages from four different experiments.

**Figure 2:**
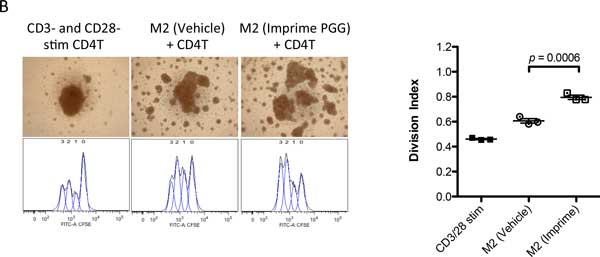
**M2 macrophages derived from Imprime PGG-treated monocytes significantly enhance T-cell proliferation**. M1 or M2 macrophages prepared as described in Figure 1 were cultured with CD3- and CD28-stimulated, CSFE-labeled, autologous CD4 T-cells at a 1:10 ratio for 3 days. T-cell proliferation was measured at the end of the experiement by flow cytometry, and results are graphically shown as CFSE-dilution peaks as well as quantitatively reported as division index (the average number of cell divisions a a population has undergone). Shown here is a representative assay with MS macrophages from three different experiments.

**Figure 3:**
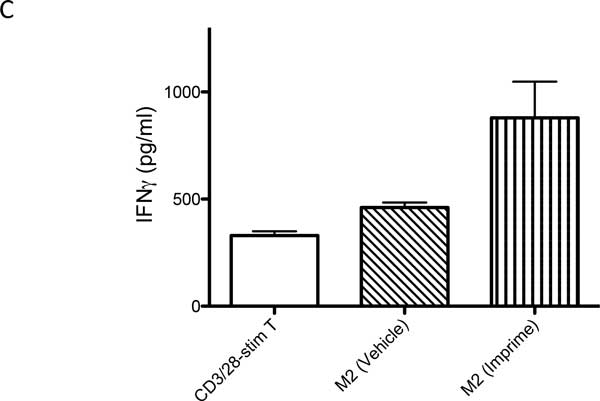
**M2 macrophages derived from Imprime PGG-treated monocytes enhance IFN-γ levels by ELISA**. Shown here is representative data from two experiments for T-cells co-cultured with Imprime PGG-treated M2 macrophages.

Monocytes enriched from IPGG- or vehicle-treated whole blood were cultured for six days in media containing GM-CSF or M-CSF to induce differentiation to M1 or M2 macrophages, respectively. Cells were then evaluated phenotypically for a panel of markers (including HLA-DR, CD163, CD206, CD209, CD80, CD86 and PD-L1) and functionally for the ability to drive CD4 T-cell proliferation and Th1 polarization. IPGG pretreatment did not affect M1 but did affect M2 as evidenced by down-modulation of CD163 and the ability of the cells to enhance CD4 T cell proliferation with a concomitant increase in interferon gamma production (Figures 1,2,3,). These changes were only observed in cells of individuals with high ABA levels.

## Conclusions

IPGG has the potential to affect M2 to M1 repolarization and drive Th1 polarization. This engagement of the adaptive, along with the innate, immune system suggests that exploration of IPGG in combination with immunosuppression-relieving anti-cancer agents is warranted.
